# Progress in the Determination of Resorcinol Using Electrochemical Method

**DOI:** 10.3390/bios15110723

**Published:** 2025-11-01

**Authors:** Chellakannu Rajkumar, Khursheed Ahmad, Shanmugam Vignesh, Tae Hwan Oh

**Affiliations:** School of Chemical Engineering, Yeungnam University, Gyeongsan 38541, Republic of Korea

**Keywords:** resorcinol, phenolic compound, environmental pollutant, electrochemical sensor, biosensor

## Abstract

Phenolic compounds such as resorcinol (RS) have negative impacts on aquatic life, the environment, and human health. Thus, it is necessary to develop sensing devices for the monitoring of RS. The electrochemical method is one of the most significant approaches for the determination of toxic substances. In electrochemical methods, electrode modifiers play a vital role and affect the sensing performance of the electrochemical sensors. Thus, the selection of efficient electrode material is of great importance. In recent years, various electrode modifiers such as graphene, metal–organic frameworks (MOFs), MXenes, metal oxides, polymers, and composite materials have been extensively used for the fabrication of RS sensors. In this review, we have summarized the reported electrode modifiers for the fabrication of RS electrochemical sensors. Various electrochemical sensing techniques, including differential pulse voltammetry (DPV), square wave voltammetry (SWV), amperometry (Amp), cyclic voltammetry (CV), and linear sweep voltammetry (LSV) have been discussed. This review provides an overview of a large number of electrode modifiers for the determination of RS. The limitations, challenges, and future perspectives for RS sensors are discussed. We believe that the present review article is beneficial for the scientific community and electrochemists working on the construction of RS sensors.

## 1. Introduction

Phenolic compounds are widely used in various industries such as cosmetics and pharmaceuticals [1,2]. Phenolic compounds are considered to be hazardous materials which have negative and toxic effects on human health, aquatic life, and the environment [3,4]. Particularly, resorcinol (RS) is utilized in plastic industries, organic synthesis, rubber, food additives, and pharmaceutical industries [5,6]. RS can also be used as a disinfectant and antiseptic for skin diseases due to its antifungal, antibacterial, and keratolytic properties [7,8]. It is understood that RS is one of the phenolic compounds which has pernicious effects on water quality and the environment [9]. Additionally, it can be stated that RS may also cause skin dryness, itching, rash, and eye irritation when in direct contact with human skin [10,11]. The inhalation of RS may also be responsible for other health issues such as shortness of breath and coughing [7]. A higher concentration of RS is also responsible for fatigue and methemoglobinemia [12]. Long-term exposure to RS may also damage the liver, human nervous system, and kidneys [13]. RS is also considered to be a carcinogenic phenolic compound which has genotoxicity and low biodegradability [7]. Therefore, researchers are paying more attention to the development of detection techniques for the monitoring of RS in environmental samples. As per the reported literature, it can be summarized that conventional methods such as fluorescence [14], surface plasma resonance [15], spectrophotometric [16], spectrofluorimetric [17], flow injection chemiluminescence [18], and gas chromatography [19] were used for the determination of various analytes. Unfortunately, these conventional techniques have their own limitations such as being time consuming, having slow processing for the detection process, high cost, and required pre-treatment processes [20,21]. Therefore, it is required to find a low cost and simple sensing technology method for the monitoring of RS with high sensitivity and selectivity.

Recently, the electrochemical method has attracted researchers globally, due to its excellent sensitivity, selectivity, simplicity, pre-treatment free, low cost, and benign fabrication and working mechanism for the determination of RS. For electrochemical techniques, it is required to decorate the active surface of the electrode with nanostructured, high specific surface area and conductive materials. The fabrication of the electrode may involve simple modification approaches such as the drop-casting method. Electrodes are generally modified with metal oxides [22], carbon-based materials (such as graphene and carbon nanotubes) [23,24], metal–organic-frameworks (MOFs) [25], polymers [26], layered double hydroxide (LDH), metal sulfides [27], and composites materials [28] using simple modification processes. Previously, various cost-effective materials were used for the development of the RS electrochemical sensor [29,30]. We believe that it would be useful to compile the recent progress in the fabrication of electrode materials for the construction of RS electrochemical sensors.

This review article compiled the recent developments in electrode materials design, including metal oxides, MOFs, COFs, ZIFs, MXenes, and other hybrid composite materials. This study systematically compares the electrochemical performance of the reported RS sensors using various sensing techniques. In addition, selectivity, repeatability, reproducibility, real sample recovery, and stability of the RS sensors have been reviewed. The limitations and future perspectives for RS sensors are also discussed. We believe that the present review article may benefit researchers to design and develop the next generation electrochemical sensors for environmental pollutant detection.

## 2. Progress in RS

### 2.1. Metal Oxide-Based Materials

**Figure 1:**
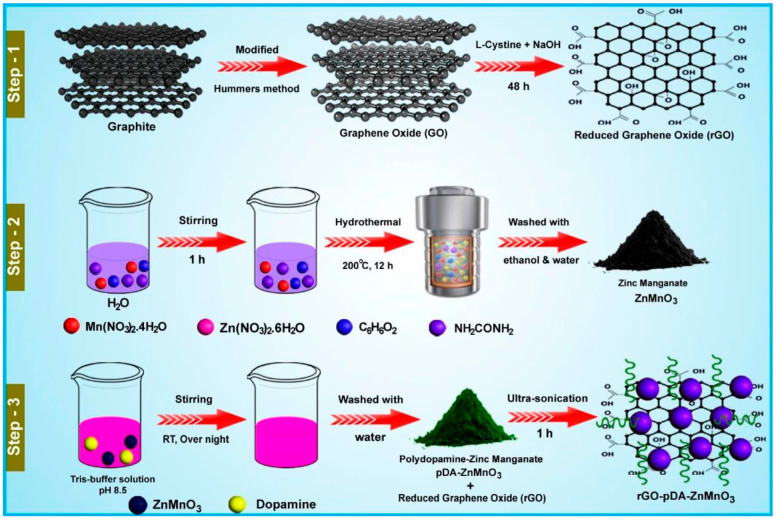
Schematic representation of the preparation of rGO-pDA-ZnMnO_3_. Reprinted with permission [36].

It is well understood that manganese dioxide (MnO_2_) is one of the semiconducting metal oxides which have decent electrocatalytic properties. However, low conductivity of MnO_2_ is the challenge for its application in electrochemical applications. In this context, MnO_2_ was incorporated with a conducting polymer, i.e., polyaniline (PANI), employing benign synthetic protocols [31]. The obtained sample exhibits that PANI comprises a nanofiber-shaped surface morphology and is incorporated with MnO_2_. The PANI/MnO_2_ composite was used as a catalyst for the determination of phenolic compounds using cyclic voltammetry and differential pulse voltammetry (CV and DPV). The PANI/MnO_2_ composite exhibits higher catalytic activity for RS detection compared to the pristine PANI or MnO_2_-modified electrode. The improved catalytic activity of the PANI/MnO_2_ composite may be attributed to the presence of synergism and the uniform distribution of MnO_2_ within the PANI framework. The fabricated electrode showed decent sensitivity and stability for RS detection. Zinc oxide (ZnO) is also a semiconducting metal oxide which has excellent catalytic properties. The cabbage-like ZnO nanostructure was prepared using the hydrothermal method and explored as catalyst for the construction of an RS sensor [32]. The surface morphological studies revealed that ZnO consists of cabbage-shaped structures with a hexagonal wurtzite phase. The proposed RS electrochemical sensor exhibited a sensitivity of 1.98 μAμM^−1^cm^−2^ and limit of detection (LOD) of 5.89 μM. It is clear that metal oxides are promising catalysts for the electrochemical determination of RS. Therefore, titanium dioxide (TiO_2_) nanoparticles (NPs) were also adopted as electrode modifiers for the development of RS sensors [33]. The TiO_2_ NPs were mixed with graphite powder and paraffin wax to fabricate the electrode for RS detection. The TiO_2_-modified carbon paste electrode (CPE) exhibits decent electrocatalytic behavior for RS detection. The authors used the linear sweep voltammetry (LSV) method for the detection of RS and obtained an LOD of 0.001 μM with acceptable recovery of 94% to 101.1% in tap water samples. It was also found that the detection of RS involves a diffusion-controlled process. The electrochemical activity of TiO_2_ for RS detection was also improved by preparing silver (Ag)-doped TiO_2_ [34]. The Ag-TiO_2_ functionalized guar gum (FGG)-based electrode was used as an electrochemical sensor for the determination of nitrite and phenolic compounds. The Ag-TiO_2_/FGG-modified electrode exhibited an LOD of 0.0776 μM and a linear range of 0.6 µM to 300 µM for RS detection. The enhanced performance was attributed to the improved electrochemically active surface area and catalytic activity. Lanthanum-based oxides such as lanthanum oxide (La_2_O_3_) NPs were incorporated with snowflake-like copper sulfide (Cu_2_S) nanostructures [35]. The obtained hybrid composite was utilized as an electrode modifier towards the construction of an RS sensor using simple strategies. The authors used chronoamperometry (CA), DPV, and CV to characterize the electrochemical properties of the proposed composite material. The electrode material was coated on a glassy carbon electrode (GCE) surface and optimized conditions exhibited an LOD of 0.059 μM with a linear range of 0.66 to 266.6 μM. The proposed electrode material was also effective for the detection of RS in real samples. Sasikumar et al. [36] prepared zinc manganate/polydopamine-functionalized reduced graphene oxide (rGO-pDA-ZnMnO_3_) using simple strategies, as shown in Figure 1. The synthesized material was explored for the detection of Isoprenaline (IP) and RS through electrochemical technology. It was observed that the integration of ZnMnO_3_ with rGO-pDA sheets provide abundant active sites and improve conductivity. The synthesized rGO-pDA-ZnMnO_3_ was drop-cast onto the active surface of the GCE and its electrochemical behavior for RS detection was performed using CV and DPV. The DPV studies show that RS can be detected with an LOD of 0.0071 μM and a linear range of 0.04 to 27.9 µM. The proposed electrochemical sensor also displayed excellent selectivity for RS detection in the presence of various interfering substances such as manganese (Mn^2+^), magnesium (Mg^2+^), sulfate (SO_4_^2−^), copper (Cu^2+^), calcium (Ca^2+^), dopamine (DA), glucose (Glu), 3-aminophenol (3-AP), 2-aminophenol (2-AP), mesalazine (MSZ), and 4-cyanophenol (4-CP). In addition, the presence of decent stability, reproducibility, and acceptable recovery in human urine samples, hair dye samples, and tap water samples suggested its potential for the real-time monitoring of RS.

In another previous study [37], an RS electrochemical sensor was also developed using bimetallic zinc/cobalt-based zeolitic imidazolate framework (ZIF)-derived ZnCo_2_O_4_ nanoplates as the electrode material, whereas carbon fiber cloth was used as the electrode substrate for the construction of a flexible RS sensor. The ZnCo_2_O_4_-modified electrode exhibited an LOD of 0.15 μM for RS detection with a linear range of 2 to 500 μM. This interesting performance of the proposed RS sensor may be ascribed to the presence of improved catalytic activity and surface properties of the ZIF-derived ZnCo_2_O_4_-based electrode. Arpitha et al. [38] also adopted a ZnO/cobalt oxide (Co_3_O_4_) composite as the sensing material for the determination of RS. The authors used a simple co-precipitation method for the preparation of the sensing material. The ZnO/Co_3_O_4_ material-modified electrode showed improved catalytic activity for RS detection with an LOD of 2.92 µM and a linear range of 10 to 60 µM. This study also reported decent stability and selectivity for RS detection which may be attributed to the synergistic effects between ZnO and Co_3_O_4_. These studies revealed that metal oxides are promising materials for the determination of RS. Therefore, bismuth tungstate (Bi_2_WO_6_) was also synthesized using a simple wet chemical co-precipitation method [39]. The presence of synergistic interactions between Bi and W improve the interactions between the fabricated electrode and RS. The Bi_2_WO_6_-modified electrode was capable to detect RS with a linear range of 20 µM to 5 mM and an LOD of 4.3 µM. The authors also found that the proposed electrode had several advantages for RS detection in terms of reproducibility, acceptable recovery in real samples (tap water), and stability. This work suggested the potential role of a Bi_2_WO_6_-modified electrode for electrochemical sensing applications. It suggested that a Bi_2_WO_6_-modified electrode may be explored for the development of sensors for environmental monitoring. In another study [40], yttria (Y) and scandia (Sc)-modified zirconium oxides (ZrO_2_) were prepared for the construction of dihydroxybenzene (DHB) isomers sensors. The prepared material was coated on screen-printed carbon electrodes (SPCEs) for the construction of the RS sensor. The authors used the square wave voltammetry (SWV) technique for the monitoring of RS. The electrochemical studies revealed that ZrO_2_-10Sc/SPCE has better electrocatalytic activities for RS detection. Therefore, an LOD of 0.00561 μM was obtained for RS detection with acceptable recovery of 90% to 116% in tap and mineral water samples. Tajik et al. [41] reported the fabrication of a manganese tungstate (MnWO_4_)/rGO composite through the hydrothermal method. The synthesized MnWO_4_/rGO was deposited on the surface of screen-printed graphite electrodes (SPGEs). The MnWO_4_/rGO composite-modified SPGEs exhibited excellent recovery of RS in tap water and river water samples. A novel electrode material that consists of carboxylated multi-walled carbon nanotubes (c-MWCNTs), Co_3_O_4_ NPs, and poly-l-valine (PolyVal) was explored for the determination of RS [42]. The optimized conditions revealed that the fabricated sensor was capable of monitoring RS with a linear range of 31 µM to 550 µM, an LOD of 7.97 µM, and satisfactory results in real samples (tap and river water). It is clear from the above observations that metal oxides may be explored for the preparation of hybrid composites with conductive materials for the monitoring of RS in real samples.

### 2.2. COF/MOF/ZIF-Based Materials

**Figure 2:**
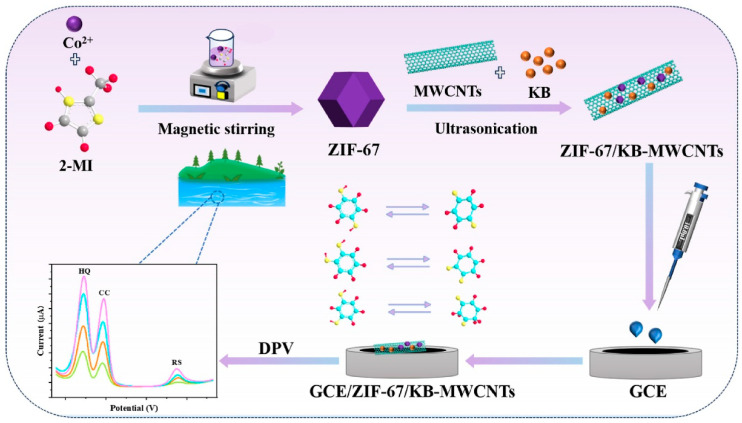
Schematic illustration of the fabrication of GCE/ZIF-67/KB-MWCNTs for electrochemical sensing applications. DPV curves show the detection of HQ, CC, and RS. Reprinted with permission [45].

It is well-known that metal–organic frameworks (MOFs) and covalent–organic framework (COFs) are high specific surface area materials with decent porosity, which makes them a promising material for the construction of electrochemical sensors. In this connection, a cobalt MOF (Co-MOF) was integrated with rGO through the electrochemical deposition method [43]. The Co-MOF/rGO composite was characterized by various sophisticated techniques and its catalytic activity was checked for RS detection. The wide linear range of 0.1 μM to 800 μM, an LOD of 0.019 μM, and decent stability for RS detection were achieved under the optimized conditions. In another previous study [44], a novel copper-based MOF (Cu-BTC-MOF/PTA (copper benzene-1, 3, 5-tricarboxylate–poly-3-thiophene acetic acid) composite was fabricated on carbon fiber paper (CFP) using facile conditions. The fabricated electrode exhibited excellent electrocatalytic properties and facilitated electron transfer for the detection of RS. Electrochemical studies revealed that an LOD of 0.008 μM can be obtained for RS monitoring. This fabricated Cu-BTC-MOF/PTA/CFP electrode also exhibited a decent linear range of 0.025 μM to 350 μM, excellent stability, and selectivity for RS detection. Ketjen black (KB)/MWCNTs/ZIF-67 was also proposed as a sensing material for the development of RS electrochemical sensors [45]. The prepared material was coated on a GCE surface and it was utilized as the sensing layer for the determination of RS and other phenolic compounds in environmental water samples. The fabrication of the electrode is described in Figure 2. This proposed electrode material offers several advantages such as improved electrochemically active surface area, abundant active sites, and improved conductivity with a three-dimensional (3D) chain structure which facilitated electrochemical reactions at the electrode surface and enhanced the sensitivity and selectivity of the RS sensor.

Arul et al. [46] reported a novel COF-based electrode material for the determination of RS. The 3,5-diamino-1,2,4-triazole-COF (DAT-COF) film was coated on a GCE surface for the monitoring of dihydroxybenzene isomers. The DAT-COF was obtained through the reflux method assisted with an electrodeposition approach. It was found that DAT-COF-modified GCEs have the potential for RS detection with good selectivity and sensitivity. The real sample analysis is also another advantage of this reported RS sensor. Another study [47] also reported the preparation of TFPB-BD-COF using 1,3,5-tris-(4-formylphenyl)benzene (TFPB) and benzidine (BD) as precursors and combined it with platinum (Pt) NPs and amino (NH_2_)-functionalized MWCNTs to construct the RS sensor. It was observed that RS can be determined with linear range of 4 μM to 360 μM, an LOD of 0.26 μM, high stability, reproducibility, and repeatability. This sensor was also selective for RS detection in the presence of interfering materials such as potassium chloride (KCl), magnesium sulfate (MgSO_4_), calcium chloride (CaCl_2_), potassium bromide (KBr), Glu, AA, bisphenol A (BPA), citric acid (CA), and p-NP. The above-mentioned electrochemical sensor also displayed reasonably good recovery of RS in yellow river water and domestic wastewater samples. The mesoporous carbon hollow sphere (MCHS)-integrated ZIF-derived Co-embedded N-doped CNTs (MCHSs/Co@N-CNTs) composite was also adopted as a sensing material [48]. Owing to the high electrical conductivity and electrocatalytic activity of the prepared MCHSs/Co@N-CNTs composite, the sensing performance of the constructed RS sensor was enhanced. The authors obtained a linear range of 20 μM to 1000 μM with an LOD of 4.21 μM towards the determination of RS (S/N = 3). The fabricated RS sensor also demonstrated promising performance for RS detection in environmental water samples. It is revealing that high specific surface area materials are promising candidates for electrochemical sensing applications.

### 2.3. rGO-Based Materials

**Figure 3:**
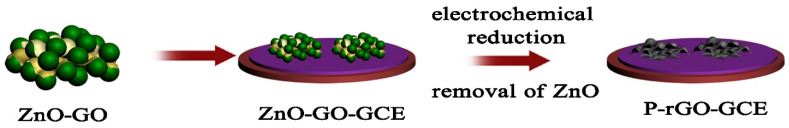
Schematic graph shows the formation of a P-rGO modified GCE. Reprinted with permission [49].

It is well known that rGO is one of the most conductive carbon materials, which has excellent electronic and surface properties. The porosity of the electrode materials also affects the electrochemical sensing performance of the sensors. In this connection, porous rGO (P-rGO) was fabricated using an electrochemical reduction approach by using ZnO as a sacrificial template [49]. The fabrication process has been described in Figure 3.

It was found that P-rGO modified electrodes exhibited an LOD of 2.62 μM and linear range of 5 μM to 90 μM for RS monitoring, which can be ascribed to the porous framework of P-rGO. The gold and palladium (Au-Pd) nanoflower (NF)/rGO composite was utilized as the electrochemical sensing material for RS detection [50]. The authors synthesized a Au-Pd NF/rGO composite through the electrochemical co-reduction approach. The fabricated Au-Pd NF/rGO composite-based electrode was used as the RS sensor which demonstrated decent electrochemical performance for RS detection. This performance may be attributed to the incorporation of Pd, which improves the catalytic properties of the fabricated RS sensor. The authors also explored the fabricated electrode for the real-time monitoring of RS in lake water, tap water, and river water samples. In another study [51], graphene (Gr) was incorporated with a conducting polymer (poly (3,4-ethylenedioxythiophene) = PEDOT) using an in situ electro-polymerization process. The scanning electron microscopic (SEM) analysis revealed that PEDOT has a 1D structure with an average diameter of approximately 200 nm. Furthermore, the PEDOT-Gr composite-modified electrode was used as the electrochemical sensor for RS detection which exhibited an LOD of 0.16 μM with good selectivity and fast response. It is believed that the presence of the 1D structure of PEDOT offers high specific surface area and synergistic interactions with the Gr-based electrode to enhance the detection of RS. In another study [52], Gr sheet-embedded carbon (GSEC) film was electrochemically activated in an alkaline potassium hydroxide (KOH) solution for the determination of RS. The authors systematically investigated the activation process and revealed that Gr sheets within the carbon matrix underwent corrosion during activation process which resulted in the formation of defective Gr edge and carbonyl functional groups at the carbon surface. The corroded Gr edge provides more active sites which enhance the electrochemical detection of RS. The linear range of 0.2 μM to 400 μM and LOD of 0.05 μM were observed for RS detection. Another research study [53] also reported the use of a poly (adenine)-modified graphene paste electrode (PAMGPE) as the electrochemical sensor for the determination of RS and its isomers. It can be stated that carbon-based materials are promising electrode materials for the development of electrochemical sensors. Nejad et al. [54] also reported the modification of CPE with GO/poly (amidoamine) dendrimer third generation, GO/G3-PAMAM) nanocomposite and an ionic liquid (IL) for the determination of RS. The fabricated GO-PAMAM/ILCPE was found to be a promising electrochemical sensor for the monitoring of RS in real samples. The rGO was also obtained using electroreduction of GO on the GCE surface [55]. The authors also utilized Pd as cocatalyst and a constructed electrode (Pd/rGO/GCE) was adopted as the electrochemical sensor. The proposed RS sensor exhibited decent recovery of RS in real water samples. The authors also obtained an LOD of 0.07 μM with a linear range of 0.1 µM to 50 µM. This enhanced performance may be attributed to the presence of synergism between rGO and Pd which improved the electron transfer and catalytic activity for RS detection. This is clear that carbon materials, especially graphene-based materials, are efficient electrocatalysts for the electrochemical detection of RS. It was observed that rGO-based materials exhibited good catalytic activity for the electrochemical detection of RS. This may be ascribed to the superior conductivity, high specific surface area, and surface structural properties. The porous rGO, Au/Pd nanoflower/rGO, and polymer- or metal-doped rGO hybrids have displayed improved electrocatalytic activity and a reasonably good LOD, which may be attributed to the presence of synergistic interactions. The synergistic interactions between rGO and incorporated materials such as metal oxides or polymers enhance the charge transfer kinetics and provide active sites for redox reactions. These reports indicated that rGO-based composites exhibited higher electrochemical performance for RS detection compared to the pristine materials.

### 2.4. CNT-Based Materials

**Figure 4:**
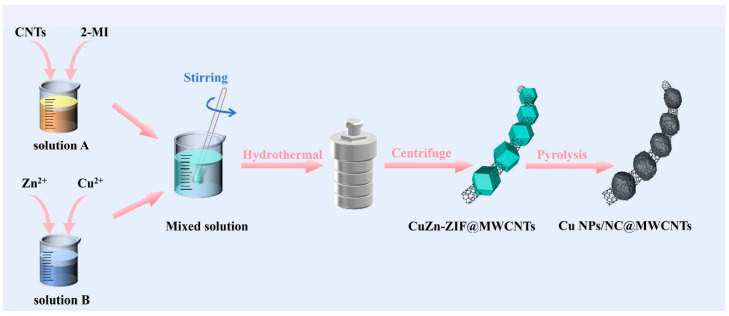
Schematic illustration of the fabrication of the Cu NPs/NC@MWCNTs composite. Reprinted with permission [59].

CNTs are the derivatives of carbon materials which offer several advantages such as decent conductivity, reasonable conductivity, and catalytic properties. The MWCNTs were modified with carbon dots (CDs) for the fabrication of nafion/MWCNTs/CDs/MWCNTs architecture towards the monitoring of RS [56]. The amide-functionalized MWCNTs were strongly linked to the CDs via electrostatic interactions and formed composite materials. This prepared composite was explored as both a catalyst and electrode material for RS detection in real samples. DPV analysis revealed that current response linearly increases with increasing concentration of RS. The LOD of 0.15 μM and linear range of 3.0 μM to 400.0 μM were obtained under the optimized conditions. The surface of the GCE was also modified with MWCNT–chitosan composites to form the MWCNTs-CHT/GCE for the determination of RS using the electrochemical technique [57]. The authors found that developed sensors are efficient for the detection of RS in spiked tap water samples. Ghoreishi et al. [58] also used MWCNT films as catalysts for the monitoring of RS using the CV, SWV, and DPV techniques. The LOD of 0.49 µM and 1.1 µM were obtained using SWV and DPV technique, respectively. The long-term stability and acceptable recovery of 93% to 104% in real samples suggested its potential for real time environmental monitoring applications. In another study [59], a necklace-like N-doped carbon polyhedron/CNTs composite/Cu NP (Cu NPs/NC@MWCNTs) was prepared through the hydrothermal assisted method, as shown in Figure 4.

It was found that presence of distinctive necklace-like porous architecture and the synergistic effects between ZIF-derived N-doped carbon polyhedra and MWCNTs may improve the electronic and surface properties of the obtained composite material. The prepared material also demonstrated excellent selectivity and sensitivity for the determination of RS using the LSV method. The LOD of 0.10 µM was obtained for RS detection with reasonable recovery of RS in environmental water samples. As mentioned in the above reports, MCHSs/Co@N-CNTs/GCE exhibits a wide linear range of 20 to 1000 µM for RS detection. It suggests that CNT-based materials are promising electrode modifiers for the determination of RS. The CNT-based materials exhibit synergistic interactions and improved conductivity/surface area which may facilitate electron transfer. Therefore, enhanced sensitivity and selectivity could be observed for the monitoring of RS in environmental samples. However, some limitations such as aggregation of the CNTs and reproducibility of the RS sensors based on CNT materials can be overcome in future studies. It is also worth mentioning that biomass-derived carbons or carbon-based materials show various advantages such as use of minimal toxic reagents, low energy, and environmental friendliness. However, physicochemical properties of the biomass-derived materials are influenced by precursor composition. This may limit the reproducibility and control over the structural properties of the biomass-derived materials. On the other hand, synthetic carbon or carbon-based materials such as graphene CNTs show excellent conductivity and surface properties. However, the synthesis of rGO or CNTs involves less environmentally friendly synthetic procedures. Therefore, future research may also focus on these points.

### 2.5. Metal Sulfide/Selenide/LDH/MXene-Based Materials

**Figure 5:**
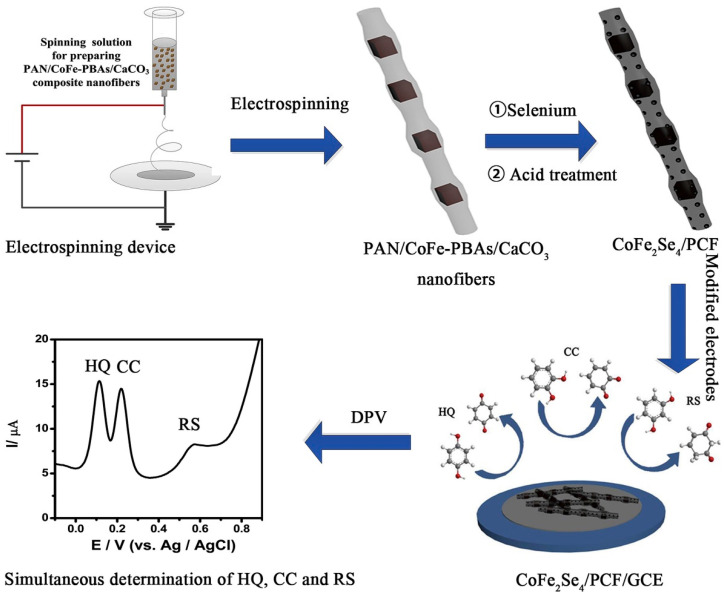
Schematic graph shows the fabrication of CoFe_2_Se_4_/PCF/GCE for phenolic compound detection. DPV curve shows the simultaneous detection of HQ, CC, and RS. Reprinted with permission [61].

In a previous study [60], it was observed that the incorporation of tungsten sulfide (WS_2_)-modified Gr (WS_2_-Gr) may show higher electrical conductivity and catalytic behavior for electrochemical reactions. WS_2_ is one of the less toxic metal sulfides which has several advantages such as cost-effectiveness and layered structure. The prepared composite may exhibit higher electrical conductivity due to the presence of synergism between WS_2_ and Gr. The WS_2_/Gr composite was coated on a GCE surface for the monitoring of phenolic isomers. The developed electrochemical sensor shows high electrocatalytic properties towards the determination of RS using the DPV technique. The WS_2_/Gr composite-modified electrode demonstrated higher current response for RS detection compared to the bare GCE, which is ascribed to the excellent catalytic properties and synergism of the prepared electrode material. The real sample studies suggested that the WS_2_/Gr composite is one of the promising and efficient materials for the development of RS electrochemical sensors for environmental monitoring. The cobalt–iron selenides incorporated porous carbon nanofibers (CoFe_2_Se_4_/PCF) were synthesized using facile conditions [61]. The electrode’s fabrication has been illustrated in Figure 5.

The obtained CoFe_2_Se_4_/PCF composites possess a 3D network structure which may provide efficient conductive pathways to enhance electron transfer. The electrochemical performance of the CoFe_2_Se_4_/PCF-modified GCE was checked for the determination of RS. This proposed electrochemical sensor exhibited wide linear ranges of 5 to 350 μM and an LOD of 1.36 μM for RS detection. Further studies also show that fabricated electrodes are highly selective, stable, and sensitive towards RS. In last few years, layered double hydroxide (LDH) and MXene materials have attracted researchers due to their excellent conductive properties, unique structure, and catalytic behavior for electrochemical redox reactions. In this connection, CoFe LDH was prepared using the electrochemical-assisted synthetic method [62]. The synthesized CoFe LDH exhibits decent electrocatalytic properties and higher electrical conduction, as confirmed by the electrochemical impedance spectroscopy (EIS) method. The CoFe LDH-modified electrode also demonstrated better electrocatalytic current response for RS detection compared to the bare GCE. The improved current response may be ascribed to the larger electrochemically active surface area and enhanced electron transfer process. The authors found that the current response of the CoFe LDH-modified electrode linearly increased with increasing concentration of RS using DPV technique. The LOD of 0.005 µM was achieved with excellent stability, reproducibility, and selectivity for RS detection. This sensor was also efficient for RS detection in river water samples which indicated its potential for real-time monitoring. Zhang et al. [63] reported the formation of FeCu MOF-919 on layered titanium carbide (Ti_3_C_2_T_x_) MXene to for the hybrid composite. It was found that the presence of Ti_3_C_2_T_x_ MXene reduced the aggregation of FeCu MOF-919 and provided electrochemically active sites for the determination of RS. The FeCu-MOF-919/Ti_3_C_2_T_x_-based electrode shows an LOD of 0.08 μM, high stability, reproducibility, linear range of 0.5 μM to 152.5 μM, and sensitivity of 0.23 μAμM^−1^cm^−2^ for RS detection. It is clear that MXene has excellent conductivity and presence of synergism between MOFs and MXene to facilitate electron transfer and enhance the detection of RS. However, preparation methods for MXene are not environmentally friendly.

### 2.6. Other Materials for RS Detection

**Figure 6:**
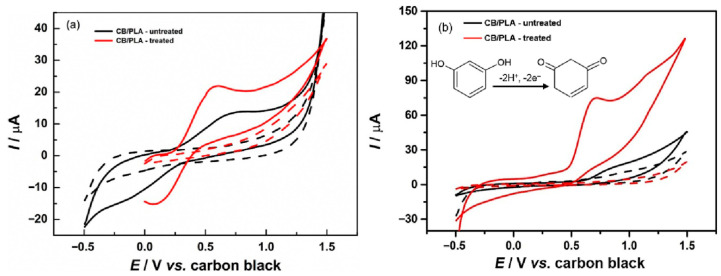
CV curves of CB/PLA before (black data) and after electrochemical treatment (red data) in the presence of (solid lines) and absence (dashed lines) of 1 mM [Fe(CN)_6_]^3−/4−^ in 0.1 M KCl (**a**). CV curves of the same electrodes in presence of 1 mM RS (**b**). Inset of Figure (**b**) shows electro-oxidation reaction for RS detection at electrode surface. Reprinted with permission [70].

Previously, numerous materials were used for the development of RS electrochemical sensors. In this connection, a green and environmentally friendly approach was used for the preparation of a well-dispersed Gr/Au NPs composite film [64]. The fabricated film was explored for the determination of RS. The obtained results exhibited a linear range of 0.01 μM to 2 µM and an LOD of 0.0022 μM for RS sensing. The real sample studies also displayed acceptable recovery. In another research study [65], a GCE was modified with poly (3-thiophenemalonic acid) towards the monitoring of RS. The wide linear range of 15.6 μM to 500 μM, and LOD of 15.6 μM were obtained with excellent sensitivity, reproducibility, selectivity, and stability which makes it a suitable candidate for the selective detection of RS. The activated screen-printed carbon electrode (ASPCE) was explored as an electrochemical sensor towards the monitoring of phenolic compounds [66]. The amperometric (i-t) approach was used for the detection of RS. The ASPCE demonstrated an LOD of 0.288 μM and linear range of 1 μM to 49.67 μM with reasonable selectivity and stability. Zhang et al. [67] reported the preparation of sodium hydroxide (NaOH) nanorods (NRs) on GCE surface using electrochemical deposition method. The authors optimized various parameters such as deposition current, deposition time, and NaOH concentration. The EIS study was conducted to examine the conductive nature of the NaOH-modified GCE. The SWV technique revealed that NaOH-modified GCEs have the potential to detect RS with an LOD of 0.09 μM and linear range of 0.03 μM to1500 μM. The presence of the NR-shaped structure of NaOH enhanced the electron transfer and mechanism for RS detection at GCE surface. The ammoniated modification with PBS activation on the GCE (CA-GCE) was carried out for the determination of RS [68]. The fabricated CA-GCE demonstrated excellent electrochemical performance for RS detection in terms of sensitivity, linear range, and selectivity. The improved performance of the proposed sensor may be attributed to the presence of hydrogen bonding between the hydroxyl groups of RS, amine, hydroxyl, and carboxyl groups on the CA-GCE surface. The kinetics revealed that the sensing of RS involves adsorption-controlled processes. The LOD of 0.47 μM and linear range of 5 μM to 200 μM were obtained for RS sensing under the optimized conditions. Chetankumar et al. [69] reported that magnesium oxide (MgO) is a promising electrocatalyst for the electrochemical monitoring of RS and its isomers. Therefore, the authors fabricated MgO-modified pre-treated CPEs (MgO-MPCPE) for the detection of environmental pollutants. The MgO was prepared using a mechanochemical-assisted synthetic method. DPV analysis revealed that current response for MgO-MPCPE linearly increases with increasing RS concentration. The authors obtained a linear range of 10 μM to 80 μM, LOD of 0.25 μM, reasonable selectivity, and acceptable recovery in real samples. The carbon black and polylactic acid (CB/PLA)-based RS sensor was also developed using novel strategies [70]. The CV curves of the treated and untreated CB/PLA were obtained in presence of redox [Fe(CN)_6_]^3−/4−^ system which revealed that treated CB/PLA has higher electrochemical activity for redox reactions, as shown in Figure 6a. Similarly, treated CB/PLA demonstrated higher electrochemical activity for RS detection compared to the untreated CB/PLA, as displayed in Figure 6b. This proposed sensor delivered an LOD of 3.4 μM, satisfactory recovery in water samples, and decent selectivity towards the monitoring of RS. The reaction for the electrochemical oxidation of RS can be seen in the inset of Figure 6b.

In another study [71], low-value Longquan lignite residue was transformed into highly active porous carbons (PCs). The PCs were activated at various temperatures of 600 to 900 °C. The observations revealed that PC activated at 800 °C showed higher sensitivity for RS detection. This sensor also delivered excellent reproducibility, stability, and selectivity for RS sensing which suggested the potential of activated PCs for environmental monitoring applications. Chen et al. [72] prepared porphyrin MOFs by integrating CB with PCN-222(Fe) (PCN = porous coordination network) through the one-pot approach. The authors found that prepared materials have high specific surface area and excellent conductivity which may improve the interactions between the targeted analyte and fabricated electrode. The DPV analysis-based investigations showed that an LOD of 0.243 μM and linear range of 0.5 μM to 320 μM towards the detection of RS. The flexible electrochemical sensor for RS detection was also developed by incorporating single-atom cobalt on N-doped Gr (SA-Co/NG) [73]. The prepared SA-Co/NG exhibits a linear detection range of 0.5 to 153.5 μM and an LOD of 0.164 μM. The synergism between pyridinic N or C=O with single Co atom enhanced the detection of RS. In another study [74], a pencil graphite electrode (PGE) modified with 3-nitrobenzoic acid (PGE/p-NBA) was explored as an electrochemical sensor. DPV analysis was performed for the determination of RS using PGE/p-NBA. A high sensitivity of 3.75 μAμM^−1^cm^−2^ with linear range of 1 μM to 300 μM were obtained for RS detection. The poly (valine) modified CPE (PVLMCPE) was also explored as an electrochemical sensor for the determination of RS [75]. The authors used DPV, CV, and EID techniques to evaluate the electrochemical performance of the PVLMCPE for RS sensing. Under the optimized conditions, the proposed electrode demonstrated decent selectivity, sensitivity, and reproducibility. It is understood that laccase as a multi-copper oxidase plays a vital role in breaking down phenolic pollutants. It was reported that laccase, like nanozyme, features copper as the active site; therefore, it is limited to the utilization of copper-free laccase nanozymes. Thus, Wang et al. [76] reported the utilization of copper-free single atom ruthenium (Ru) as an alternative to laccase. It was observed that single atom Ru mimics the active center and catalytic function of laccase. The single atom Ru nanozyme-based electrochemical biomimetic sensor displayed an LOD of 0.038 μM for RS sensing. The density functional theory (DFT) study also revealed that excellent activity of the Ru nanozyme may be ascribed to the high affinity and reactions kinetics between the targeted analyte and single atomic Ru site. In a recent study [77], a GCE was modified with Au NPs and a 4-nitroaniline (4-NA) monomer followed by the electrochemical over-oxidation process in the presence of 0.1 M NaOH which yielded GCE/AuNPs/r-pNA_over-oxidized_. The GCE/AuNPs/r-pNA_over-oxidized_ exhibits excellent electrochemical performance for the monitoring of RS using semi-derivative voltammetry (SDV). The authors were capable of achieving a linear range of 0.8 µM to 500 µM and excellent selectivity for RS detection. In another previous study [78], a novel electrochemical sensor for RS detection was also proposed by using molecularly imprinted polymer (MIP) technology. This suggested that MIP-based RS electrochemical sensors may exhibit excellent sensitivity, selectivity, and stability. The electrochemical performance of the various reported RS sensors is displayed in Table 1.

## 3. Conclusions

In this review article, we have summarized the progress in metal oxides, carbon, MXene, MOFs, and hybrid composite-based materials for the monitoring of RS. RS is one of the phenolic compounds which has toxic effects on human health and environment. Extensive research studies were reported for the determination of RS using the electrochemical method. The metal oxide-based RS sensors exhibit decent stability but the presence of low conductivity of semiconducting metal oxides is major concern. To improve the electrical conductivity and electrochemical performance of the metal oxide-based RS sensors, some novel strategies such as doping, conductive carbon hybridization, or the preparation of metal oxide-based composites with MXene, etc., need to be designed and explored for the fabrication of efficient RS sensors. Similarly, carbon materials offer high specific surface area and electrical conductivity but agglomeration may affect its electrochemical performance for RS detection.

MXene-based materials are promising materials with high conductivity and surface properties with unique layered structures, but synthetic methods are not environmentally friendly. Polymers demonstrate high conductivity and reasonable performance for RS detection but their long-term stability is limited. As per the summarized reports, it was found that rGO-pDA-ZnMnO_3_/GCE exhibits excellent sensitivity of 14.4923 μAμM^−1^cm^−2^ and recovery of RS in hair dye samples. The LDHs Co/Fe-LDH/GCE and MnWO_4_/rGO/SPGE displayed an LOD of 0.005 μM for RS detection using DPV technique. However, in some cases DPV has some selectivity issues due to the presence of isomeric mixtures. The amperometry method may be a promising approach for the determination of RS sensors but the exact mechanism of RS detection at the electrode surface is not clear. Thus, a more in-depth study is required for the exact sensing mechanism for RS detection using the amperometry method. Future research may focus on the design and use of greener synthesis routes such as HF-free MXene exfoliation and low temperature carbonization, etc., for the preparation of MXenes and its composites with LDH/MOF materials. We believe that hybrid composites of MXenes and LDHs may be promising materials for RS detection. In the future, electrochemical sensors can be integrated with next-generation flexible devices and smartphone-based devices for the monitoring of RS. The machine learning technology may be utilized to optimize the electrochemical sensing performance of RS. The future studies may also focus on real-time in situ environmental monitoring of RS.

## Figures and Tables

**Table 1:** Electrochemical sensing activity of the various reported RS electrochemical sensors.

Modifiers	Sensing Method	LOD (µM)	Sensitivity	Linear Range (µM)	Real Sample Studies	Refs
PANI/MnO_2_	DPV	0.09	0.5 μAμM^−1^	0.2–100	Tap water	[31]
C-ZnO NSs	I-V	5.89	1.98 μAμM^−1^cm^−2^	0.03–3.0	-	[32]
Ag-TiO_2_/FGG	SWV	0.07758	-	0.6–300	Well water	[34]
La_2_O_3_ NP@SF-L Cu_2_S NS	DPV	0.059	-	0.66–266.6	Tap and mineral water	[35]
rGO-pDA-ZnMnO_3_/GCE	DPV	7.1	14.4923 μAμM^−1^cm^−2^	0.16–27.9	Hair dye	[36]
ZnCo_2_O_4_ NPAs/CFC	CV	0.15	15.18 μAμM^−1^cm^−2^	2–500	Lake and river water	[37]
Zno/Co_3_O_4_/MCPE	DPV	2.92	-	-	Tap water	[38]
Bi_2_WO_6_	SWV	4.3	-	20–5000	Tap water	[39]
ZrO_2_10Sc/SPCE	SWV	5.61	0.651 μAμM^−1^cm^−2^	0–100	Tap and mineral water	[40]
MnWO_4_/rGO/SPGE	DPV	0.005	-	0.01–600	Tap and river water	[41]
CoMOF/rGO paper	Amp	0.019	-	0.1–800	Tap water	[43]
ZIF-67/KB-MWCNTs	DPV	0.198	-	2–180	River and lake water	[45]
TFPB-BD-COF/PtNPs/NH_2_-MWCNT/GCE	DPV	0.26	-	4–360	Tap water, river water, and sanitary sewage	[47]
MCHSs/Co@N-CNTs/GCE	DPV	4.21	-	20–1000	Water samples	[48]
P-rGO	DPV	2.62	-	5–90	Tap water	[49]
Au-PdNF/rGO/GCE	DPV	0.7	-	2.0–100	Tap, river, and lake water	[50]
KOH-activated GSEC film	SWV	0.05	-	0.2–400	-	[52]
Pd/rGO/GC	CV	0.070	-	0.1–50	Tap, lake, and Oilfield wastewater	[55]
MWCNTs/CDs/MWCNTs/GCE	DPV	0.15	-	1–400	Tap, well and river water	[56]
MWNTs/GCE	DPV	0.49	-	1.2–190	Artificial waste water	[58]
WS_2_-Gr/GCE	DPV	0.1 μmol dm^−3^	-	1–100 μmol dm^−3^	Pond, sewage, river, and rainwater	[60]
LDH Co/Fe-LDH/GCE	DPV	0.005	-	0.0075–4.2	Lake water	[62]
FeCu-MOF-919/Ti_3_C_2_T_x_/GCE	DPV	0.08	0.23 μAμM^−1^cm^−2^	0.5–152.5	Tap water	[63]
PTAGCE	DPV	3.91	-	391–500	-	[65]
ASPCE	Amp	0.289	-	1–49.67	Hair dye	[66]
NaOH/GCE	SWV	0.09	-	0.5–1500	Tap water	[67]
MgO/MPCPE	DPV	0.25	-	10–80	Tap water	[69]
3D printed CB/PLA	SWV	3.4	0.0065 µA.µM^−1^	5–400	Water	[70]
SA-Co/NG	DPV	0.164	-	0.50–153.5	Textile waste water	[73]
PGE/p-NBA	-	0.16	3.75 μAμM^−1^cm^−2^	1–300	-	[74]
Ru SA/GFs	DPV	0.038	-	0.1–800.1	River water	[76]
GCE/AuNPs/r-pNA_over-oxidized_	LSV	0.4	-	0.8–500	Waste water	[77]

## Data Availability

No new data was generated for this study.
